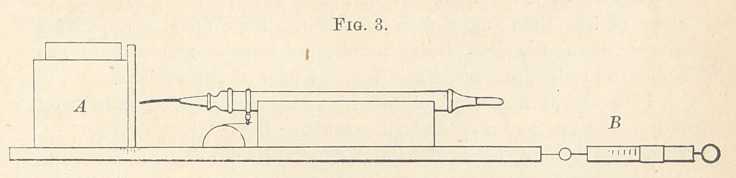# The New York Institute of Stomatology

**Published:** 1897-02

**Authors:** 

**Affiliations:** The New York Institute of Stomatology


### THE NEW YORK INSTITUTE OF STOMATOLOGY.

A meeting of the Institute was held Friday evening, November 13, 1896, at the residence of Dr. S. H. McNaughton, 63 West Forty-ninth Street, New York City, the President, Dr. Benjamin Lord, in the chair. The minutes of the previous meeting were read and approved. Dr. Davenport read the following report of the special committee, appointed at the last meeting, in the matter of the death of Dr. C. F. Ives:

Mr. President and Gentlemen,—The sad death of Dr. Charles F. Ives, which was so feelingly spoken of at our last meeting, caused the appointment of your committee, and we desire to recommend that as a society we record our sense of loss, and our appreciation of his high personal and professional qualities, by entering this expression of them upon our minutes, and by making them a part of our transactions. We naturally recall the fact that he took the greatest interest from the beginning in the organization and proposed policy of this Institute, and that he was willing, notwithstanding the discouragements of failing health and consequent inability to attend to his practice, to assume the duties of secretary in our organization. He was born in New Haven, Conn., on October 5, 1829, and received his early education in that town. In his younger years he became a telegraph operator, and his acquaintance with a dentist and his interest in the mechanics of dentistry led him at that time to conclude to make it his vocation. He became a student of Dr. Perkins, the inventor of the Perkins operating-chair, and after a sufficient training practised successively in Waterville, Watertown, and Little Falls, N. Y. He remained seven years in the latter place, coming to New York about the year 1866. He exercised unusual skill and thoroughness in his professional work, and his services were characterized by sincerity of purpose and correct judgment. He was an expert in the forming and tempering of instruments, and was fond of exercising his art in this way. In his personal intercourse he was cordial and kindly, and always ready to undertake a task or duty for those to whom he was attached. He served the New York Odontological Society as secretary for several years, and later took the same duties upon himself here at our request. It will be remembered, also, how constantly at our social gatherings around the dinner-table he was chosen cashier without a murmur on his part. He had musical ability of no mean order, and for many years was an organist in church. Although naturally reserved and retiring, his intellectual and social qualities made him a desirable comrade to those with whom he felt acquainted, as we can all testify. His latter days were sad and lonesome, yet his independent spirit did not allow him to yield to the desire of friends to entertain him in order to cheer him. He died on September 14, 1896, and his body was interred at Champion, Jefferson County, in this State.

(Signed) J. MORGAN HOWE.
S. E. DAVENPORT.

On motion, the report of the committee was accepted and ordered on file.

COMMUNICATIONS ON THEORY AND PRACTICE.

*The President.*—We now come to communications on theory and practice. Any one present, whether a member of the society or not, who has anything new or of special interest, is invited to present it at this time.

*Dr. Davenport.*—While I hardly feel like beginning, it seems to me that this very important department of our evening's programme ought not to pass. It is said that we learn more from failures than from successes; and I should like to describe one of my failures, a failure of diagnosis. Fortunately, no damage was done, though, perhaps, the next time we would not be so fortunate. A gentleman consulted me a few days ago concerning a swelling about the roots of the first right superior molar. It had every appearance of an alveolar abscess in one of its primary stages; the tooth was slightly elongated, very tender to pressure, even of the tongue; the buccal side of the gum being considerably swollen, the palatine side a little. The other teeth in the vicinity were not tender to percussion. The patient, himself a dentist, agreed with my diagnosis, that the first molar was a tooth without a living pulp; and as there were no large fillings, and no history of recent work upon it, it seemed like one of those cases of dental suicide. With a sharp bur I began to drill through a small tin filling in the anterior part of the crown of the tooth; but as soon as the bur pierced the filling and entered the dentine, the patient winced, and not from the pressure. In short, the pulp was alive and apparently in a normal condition. There had been no error as to which tooth was the cause, or at least the centre of the trouble, for the others were perfectly comfortable to all tests. The patient is, perhaps, twenty-five years of age; gums healthy; no considerable amount of tartar in the mouth, and yet the history of that tooth for the last two days has caused the patient and myself to come to the conclusion that this is a calcic abscess, and that the pulp of the tooth is in no way involved. A very fine scaler introduced on the posterior side of the second buccal root discovered a few nodules of tartar a short distance above the normal margin of the gum, and becoming rather persuasive with the scaler, a little pus was finally reached. Hydrogen dioxide being used to syringe at that point, relief has come slowly. That was the only point about the tooth where any tartar could be found. This is the third case in my experience where teeth with every possible indication of ordinary abscess from a dead pulp have been found to have calcic abscesses. I have seen very little about this condition in dental literature.

*Dr. McNaughton.*—Dr. Kirk has recorded one case in his own mouth.

*The President.*—We will now pass to the reports of the standing committees. The first is the Committee on Operative Dentistry. Dr. Bogue, the chairman, is not present; but it is well understood, I presume, that members of the committees may make individual reports, and not depend wholly upon the chairman to make a report for the committee.

*Dr. George A. Wilson.*—Might I suggest, Mr. President, that Dr. Allan be called upon to report the result of the use of the cataphoric instrument in obtunding for the removal of a live pulp? I had an invitation, but was unable to witness the operation.

*The President.*—If Dr. Allan has anything of interest in regard to operative dentistry we will be pleased to hear it.

*Dr. George S. Allan.*—The case Dr. Wilson alludes to was a right superior lateral. The lady came to me with one of those hideous gold caps over the tooth, and wished to have it changed. On taking off the cap I found that the pulp was alive, and the crown badly decayed.

It was impossible to put a porcelain-faced crown on the root, owing to its prominence, without destroying the pulp, and there was every indication that the pulp had commenced to destroy itself. I applied citrate of cocaine with the cataphoric current, using eight or ten cells of the Van Woert apparatus. I continued the application for about fifteen minutes, and then, as it was getting late, I cut the operation short, although I felt quite certain that I had not obtained complete insensibility. I found that the pulp was so nearly insensible to pain that two-thirds of it was removed without the patient wincing or paying any attention to it. Then, rather than continue the operation, as the patient was tired and nervous, although she acknowledged she was not in pain, I applied a local obtundent and dismissed her until morning, when I finished the operation by the use of ordinary arsenical paste. Five minutes more of the cataphoric current would undoubtedly have produced complete insensibility, and the whole pulp could have been removed without pain. My success with the cataphoric current seems to me to be greater every day that I use it. I do not find as much use for it in obtunding sensibility as some others do. I have noticed one or two peculiarities: in one case I applied the current and the patient was delighted,—said she could come now to the dental office and not feel worried, all fear of the operations having been removed. At the next operation I supposed, of course, that she would want the cataphoric current; and she said, "Doctor, is it all ready?" To which I replied, "Yes, it is all ready." "Well," she said, "never mind; keep right ahead, and if necessary we will have it later." But, although she has had work done several times since, she has not found it necessary to have the cataphoric current. I think the moral influence, however, was very satisfactory.

While we are speaking of the cataphoric current I wish to exhibit this card of specimens, and at the same time request the secretary to read an article which has induced me to write to Dr. Bethel, and from whom I have obtained the privilege of exhibiting his specimens. The idea illustrated here is the use of the cataphoric current in forcing nitrate of silver into the dentine of the roots of dead teeth. The subject is exceedingly interesting, and the success that Dr. Bethel has met with, as exemplified in the specimens he has so kindly sent here for our examination, I think warrants my asking you to listen to this article. I will therefore call upon our secretary to read the article, and then exhibit the specimens for your examination.

*The President.*—Gentlemen, you will give your attention to this article presented by Dr. Allan.

The secretary read, from the *Dental Digest* for October, 1896, an article entitled "Lining Root-Canals," by S. P. Bethel, D.D.S., M.D., of Kent, Ohio; also a letter of Dr. Bethel to Dr. George S. Allan, explaining the exhibit of specimens.

*Dr. Allan.*—I wish to pass around this card of specimens that Dr. Bethel has so kindly sent me. I think it will pay all to examine them with the magnifying glass carefully, especially No. 4; and notice, also, in No. 2, which was operated on in the mouth, that the crown of the tooth is not discolored perceptibly; showing that there need be no disfigurement from the use of nitrate of silver. These specimens are very instructive as showing what the cataphoric current can do in this direction. I hope to carry on my correspondence with Dr. Bethel, and I feel very certain from the tone of his letters that he will give us the benefit of his future experiments. It will be noticed that the nitrate of silver has penetrated about one-thirty-second of an inch into the substance of the dentine, completely closing the mouths of the dental tubules and preventing the ingress of bacteria.

*Dr. W. St. George Elliott.*—I happened to be present when this paper was read, and in justice to the profession it appears to me that we ought to get at all sides of the story. The discussion, particularly the part taken by Dr. Taft and Dr. Abbott, was most interesting. While they did not deny the efficacy of the process, they contended that there were certain objections to it, and that precisely the same results could be obtained in the ordinary way; many dentists having used nitrate of silver for many years for that purpose.

*Dr. Allan.*—I have no objection to the discussion of this paper being read, if the members would like to hear it. I had not the slightest idea of preventing the other side from being heard, but to save time suggested the reading of the article itself.

*The President.*—Perhaps, as Dr. Elliott was there, he can give us some account of the discussion which may be sufficient for us.

*Dr. Elliott.*—Personally, I do not think that the opponents of Dr. Bethel made out a strong case. Dr. Abbott seemed to be opposed to cataphoresis in general. Dr. Taft stated that in his own experience he found that the staining complained of was most superficial, and could be readily removed by scraping. This is not in accord with my own observation. While in London, I had a patient who had very protruding canines, and a dentist—I am sorry to say it was an American dentist—had treated the case in a very heroic manner, had excised a portion of the canines, ground off all the projecting portions, almost exposing the pulp in doing so, and naturally made the teeth exceedingly sensitive. He then applied nitrate of silver; and the teeth were very badly discolored when I saw the case five years afterwards.

*Dr. C. B. Parker.*—In relation to nitrate of silver; three weeks ago a physician came to my office suffering with a congested pulp. I removed the filling and applied the cataphoric current for thirty minutes, using cocaine, thinking that I could remove the pulp then, but I found it just as sensitive as ever; the cocaine apparently had not had any effect, and the man was suffering so intensely that for the moment I did not know what to do. I made up my mind that cocaine was not going to give me the result that I wanted, and I applied a solution of nitrate of silver, expecting only a temporary effect. I applied the current for six minutes. After two or three minutes he said the pain had subsided, but I thought I would continue it a few minutes longer. I did so, and after six minutes I tested the pulp, and was able to remove it completely without pain.

*Dr. McNaughton.*—What strength of nitrate of silver did you use?

*Dr. Parker.*—I make the saturated solution.

*Dr. Elliott.*—I would like to say that on theoretical grounds nitrate of tin would be preferable to nitrate of silver. Speaking of cataphoresis and cataphoric instruments, I have, like the most of us, paid a good deal of attention to the matter the last few months. My son has been connected with several of the companies, and has had a very extensive experience during the summer, making some two hundred and fifty demonstrations on patients, including pulp-extractions, and there were several interesting points brought out in his experience. One was the fact that cocaine is not to be relied upon when the solution is more than two days old. The second point was that eucaine is found to be in many cases preferable to cocaine; and a combination of eucaine and cocaine seems to be very efficient. Another point was that in single-rooted teeth the result is always more favorable. If the case is not successful it is probably owing to some defect in the insulator of the current.

With the molar pulps there is more difficulty. Of course, the current will not divide itself and pass down through the different roots of a tooth, but will naturally take the channel that has the least resistance; and the result is that one root may be anæsthetized, while the others are not, and the process has to be repeated, first passing the electrode down into the canal which is most accessible, and afterwards following with the others; and in that way it is perfectly successful.

*Dr. Parker.*—In over thirty attempts to remove pulps cataphorically, the one I have just referred to is the only one that gave me the slightest trouble, using a solution of cocaine, and that tooth had been giving the patient a great deal of pain for about ten days.

*Dr. Allan.*—Some time ago I saw an article on gold filling, with special points for hand-pressure. I think I had better read it hastily, and then pass around these instruments, so that all can see them and discuss their merits.

Dr. Allan read an article entitled "Gold Filling, with Special Points for Hand-Pressure," by A. D. Barker, D.D.S., of Grinnell, Iowa. Read before the Iowa State Dental Society, May 6, 1896.

*Dr. Allan.*—I have a set of points made on this same principle. The S. S. White Company now has the patterns in the Philadelphia depot, and they have kindly loaned them to me for your inspection. There is no doubt but what there is a principle involved in the shaping of these points, and I think a correct one. Take a round cavity and attempt to pack gold perfectly against the walls, with any but very fine points, and the result is that the gold will not be packed homogeneously. That is easily demonstrated. There is an old saying that one cannot put a round man into a square hole, and certainly one cannot pack gold into a round-shaped cavity with square-shaped plugger-points and have a perfect and uniformly condensed plug. The idea that Dr. Barker has is that the cavity, being shaped like the end of the finger, the filling-instrument should be of a similar shape, so that when the gold covers the point of the instrument the gold and the instrument will fit the wall and the gold be packed evenly at that point. The instrument being serrated, of course one gets the benefit of the mechanical principle of the wedge, as well as being able to use the points to burnish down the gold. I would like to call attention particularly to three forms, which please look at with the magnifying-glass. One is round ended and another is flat. The serrations in one case run lengthwise of the point, while in the second they are cut crosswise. The third is simply a fine burnisher. The handles are just large enough to fill the hand, and the short shank gives a power that one cannot appreciate without use. I think these instruments and the principle in them constituted are worthy of much attention.

*Dr. Elliott.*—Mr. President, it has been remarked that it frequently happens that inventions of the same general character are brought out by different men in different places at or near the same time. Dr. De Trey, an old friend of mine, recently brought out a set of instruments of the same general character as these exhibited by the last speaker. The details are not the same, but the principles involved are, and he considers them essential in the use of solila gold. I imported some during the summer, and also secured a copy of the American patent to find out what the gold really consisted of. As far as I could tell it is nothing more than mat gold; not deposited upon a sheet of foil, but merely mechanically locked into a sheet of foil. The objection to the S. S. White mat gold is the great waste in working: the pieces do not hang readily together in the continuous filling of a cavity. I am under the impression that De Trey's gold is practically the same thing. The crystals are finer, however, and seem to require less effort to condense. The principles adopted in filling are the same apparently as those introduced by Dr. Royce and subsequently by Dr. Barker.

*Dr. Davenport.*—Dr. J. F. Adams, of Worcester, Mass., has recently designed a chin-rest, and I have the principal parts here to exhibit to-night. The portion which I have not brought is the malleable iron socket which is attached to the chair, of no matter what make, by two screws running through the screw-holes already existing in the iron frame of the chair. The socket referred to receives this nickel-plated standard, and this standard, having a ball-and-socket joint, receives the main shank of the chin-rest proper. The portion of the apparatus upon which the patient's chin rests is secured by a ball-and-socket joint, and also has a covering, which can be made an air-cushion by the introduction of air. The two ball-and-socket joints and one single clamp make the apparatus almost universal in its application. I should like to say, Mr. President, that I have possessed one of these rests for several weeks, and while I have not used it many times, it always being difficult for me to adopt new appliances, it has been of distinct service in several cases where important and lengthy operations were being performed upon the lower teeth. There are some patients who, whether from age or nervousness, after sitting for a little time with all the paraphernalia that we subject them to in position, are troubled with a most disagreeable shaking or trembling of the lower jaw, which makes the operation much more difficult because of the lack of security and solidity of the object upon which we are working. Aside from the help in that way to the operator, this affords a distinct and comfortable rest for the patient. It is also of service when operating for young children, who very often exhibit a marked tendency to slip down upon the foot-rest. I should be very pleased to exhibit this instrument attached to the chair in my office.

*Dr. Elliott.*—Mr. President, I would like to make some remarks on the subject of mallets. Of course, we all have our opinions, and, unfortunately, we are too much given in public meetings to giving our opinions without giving the proof for the reason that is within us. In the matter of mallets there are a number of opinions, one operator preferring one kind and another preferring another. It seems to me that we are able to bring the question down to something like a scientific basis; there ought to be but one way of doing a thing, and that the best way, and the best way ought to be generally adopted. That leads me to remark that my own belief in the conservatism of the profession is so great that if there were demonstrated here, for example, a process of condensing gold which would meet every requirement, which would be scientific, accurate, and rapid, there would not be a single person who would adopt it, all continuing to do just as they had been doing. Years ago a gentleman in London said to me, My process of filling with gold is exceedingly rapid; I use the hand-mallet and mallet for myself; I have a young lady assistant who conveys the gold, and I claim to be able to do work quicker than it can be done in any other way and equally well. I immediately challenged this gentleman to a trial test in filling. In the course of several weeks half a dozen other gentlemen joined us. Each operator had the choice of his own method, was to take his own time, and keep account of the time consumed, and to perform the operation in his own way. One malleted for himself, one had an assistant to mallet for him, there were three or four who used electric mallets, and one who used the pneumatic mallet. The operations were performed, of course, out of the mouth, and in similar cavities as far as possible to prepare them, cavities in molars that were made difficult of access by the presence of other teeth, all mounted in plaster. The teeth to be operated upon were prepared by a third person and given out by lot, and the operators returned the work performed to an umpire, who made a thorough examination of it. Tests were made of the surfaces for solidity, polish, homogeneousness as far as it could be recognized, the force with which the plug was held in the tooth, and for other points of more or less importance, and the curious fact

Fig. 1.

which came out was that the shortest operation was performed by a man who malleted for himself. He took an hour and a half to perform the operation, while the one who had an assistant to mallet for him took an hour and three-quarters, and the electrics took about five hours each. (I have not the report by me.) The work performed by the electrics did not prove to be as good as that of the gentleman who had an assistant, and who took the prize. I merely mention this incident on account of the fact that there was not a single man, as far as I know, who changed his mode of operating as a result of the trial.

Now, in regard to mallets, I have made a series of experiments, with a view to getting some positive data in regard to the force required. I have made a number of forms of pneumatic mallets. This one was made in London, and carries a large flyer of considerable weight. Then I found that the majority of patients are somewhat curious to know what was the force used, how the gold was malleted by something which was inside. For the purpose of satisfying that curiosity I made it of glass (Fig. 1), recognizing the fact also that glass has a very material advantage in causing less friction. Then I had to get over the difficulty in connection with glass, the liability to breakage, and that I succeeded in overcoming by receiving the blow on a spring. I have reduced the bellows to its simplest form. (Fig. 2.) It con-

Fig. 2.

sists of two pieces of spring brass, two hemispheres, one passing inside the other, which is covered with rubber dam, held by a wire ring, which enables one to change it in a moment. I have used it for two years; the rubber has to be changed once in three or four months. The pneumatic mallet fills a certain field which is not filled by any other instrument. It does not compare, for instance, with the hand-mallet for universality. The class of cavities for which I prefer the pneumatic are those anterior cavities, in what are sometimes, but erroneously, called the oral teeth. There the pneumatic mallet seems to work with far more delicacy of force than any other; is even more delicate than hand pressure.

In some experiments that I have made in regard to the penetrating power of different forms of mallets, I found that by taking an ordinary automatic (Fig. 3),—the well-known Abbott instru-

Fig. 3.

ment, for example,—the average force required to operate it was two and a half pounds. The efficiency of the blow is found by multiplying the weight of the hammer by the square of its velocity. The weight in this hammer is twenty-three grammes. By the use of a heavier weight, whether it be a hand-mallet or an automatic mallet, the whole tooth must naturally be jarred; whereas with the light weight and high velocity its effect would be upon the surface of the gold and not through the body of the tooth.

If we are using soft foil the whole question is altered, as we then wish to produce an effect far beyond the surface. Many gentlemen, in speaking of the relative solidity of their work, say it is solid because it was reduced to a sheet of gold by hammering on an anvil or by rolling, forgetting the fact that it is the hammering or rolling that condenses it. The only way to test the solidity of a filling is by its specific gravity. This instrument has been designed to test the effect of different forms of blows. (Fig. 3.) *A* is an anvil weighing four and a half pounds. A pad of ten sheets of writing-paper is placed against it to receive the blow of the instrument to be tested. Using the same sharp point, it was found that with a pull on a spring-balance (*B*) of two and a half pounds the Abbott penetrated five sheets (flyer, twenty-three grammes) and moved the anvil block one-quarter of an inch, while the pneumatic with light flyer (three grammes) penetrated six sheets and failed to move the anvil even when reduced to two pounds. We are thus led to this conclusion, that in the anterior teeth, particularly when loose or painful in their sockets, it is best to use a small weight and high velocity, as it packs the gold better and does not disturb the tooth.

I have experimented a good deal with cataphoric outfits of various kinds. I prefer the street current to a battery, but must have it absolutely reliable, and believe I have accomplished it. An electrician of the Edison company was informed that the fluctuation of the current was not more than two volts in one hundred and eighteen on this section, which would make a very small change when only ten volts are being used.

As far as I know, none of the apparatus in present use will give steps of less than one-eighth of a volt. The Wheeler apparatus gives a change of one-fifth of a volt, and steps of this amount are liable to produce some sensation on the part of the patient.

I use the Willms controller, which has one hundred and eleven steps. I have had a special rheostat made. It gives five, ten, fifteen, twenty-five, thirty-five, and fifty volts, so that I can commence with five volts pressure, and in steps of one-twentieth of a volt gradually increase to fifty volts, if necessary. I take current off the main circuit with constant resistance by a shunt, pass it through the Willms milliampère metre, and thus practically get rid of any variation in the street current.

*The President.*—The Committee on Prosthetic Dentistry, Dr. Bishop, chairman, will now report.

*Dr. J. Adams Bishop.*—As the chairman of the Committee on Prosthetic Dentistry, I take great pleasure in submitting the following report:

The New York Institute of Stomatology, at its monthly meetings during the first year of its life, has had some rare cases presented to it, showing the results of treatment by mechanical dentistry or mechanical surgery, and it may be years before the like are presented again. Our report is only a review of the cases.

At the November meeting of 1895, Dr. C. O. Kimball reported and exhibited the cast of a case of fracture of the superior maxilla. Miss D., aged eighteen years, was struck with a golf-stick, breaking off the corners of two lower teeth, cutting through the upper lip, crushing in the left upper central and lateral teeth, and separating the two maxillæ at their anterior portion by nearly a line.

Owing, in part, to the extensive laceration of the lip, the jaw was not attended to until ten days after the accident. Then sectional impressions of the upper jaw were taken, the arch restored, and a splint made to fit the model.

The two sides of the jaw were drawn together and the splint applied, pushing out the left lateral and central teeth to their proper positions. Perfect union and firmness of teeth were secured, none of the teeth dying, but a slight permanent separation of the central incisors remained.

At the same meeting I had the pleasure of presenting a patient, the condition of whose mouth was the result of a sharp-pointed stick having been forced through the soft palate, displacing teeth and causing the loss of a portion of the superior maxilla, so that the mechanism of the mouth was greatly injured. The treatment was the same as for cleft palate.

Getting a good impression of the mouth, and especially of the back molars, which would give a good support, I made a plate, covering the mouth and making it air-tight.

The result was most gratifying, especially to my patient. He goes down town to business and does not hesitate to come in contact with any one, as this plate gives him the natural use of his organs of speech.

At a meeting of this Institute held at the Polyclinic College, on February 4, 1896, Dr. Dawbarn gave a lecture and surgical demonstration, as reported in the INTERNATIONAL DENTAL JOURNAL for April, 1896. Dr. Dawbarn described to you the difficulties and the magnitude of the operation he had performed upon a patient whom he exhibited there, but his patient's life depended on his performing it.

The condition of this patient, after the removal of the superior maxilla of one side, was all favorable for a healthy recovery from the operation, after which the parts had to be restored by artificial means. To enable the patient to resume his daily occupation, his voice and looks were the two things to be considered first. A good impression was obtained, and a dental fixture covering the roof of the mouth and supplying the left half of the dental arch with teeth was made. The entire right side is embraced or clasped by the plate, which is so balanced that it is worn constantly with entire comfort and scarcely betrays its presence.

One of the very important departments of prosthetic dentistry is the making of metal dies. The swedging of gold or platinum plates is a delicate thing to do well. Suppose we have a patient who needs a gold plate for the superior maxilla, which means a suction plate. The impression with plaster is the first step. I prepare the mouth for the plaster by wiping off all the saliva that forms a covering over the mouth, really making it larger.

I find by so doing that my impression is so perfect that it is with difficulty I remove it. Then I proceed, using all the care I can so as not to have a blemish upon my cast. I give the impression two coats of varnish which, of course, makes the cast a trifle smaller. Trimming the plaster cast made from that impression down very thin, I fasten it into an iron cup, which is four inches in diameter and three inches high, leaving at least two inches for the metal, hereafter described. The metal is poured into this cup as soon as the plaster is thoroughly dry.

The plaster cast is perfectly dry if no moisture condenses on the face of a mirror held over it. The plaster cast will then receive the molten metal perfectly. As soon as the metal is in the cup, chill the iron cup with cold water on the outside, so as to induce the metal to concentrate around the plaster cast. The metal for this, the female die, is composed of about two-thirds tin and one-third lead. When cold it will readily come out of the cup, and should then be thoroughly cleansed, the plaster cast—for which there is no further use—being cut away.

It is then ready for the male die, which is made of fusible metal, one-half bismuth, one-quarter lead, and one-quarter tin, so that it melts at about the temperature of boiling water. An iron ring is placed upon the female die a little larger than the impression, and the fusible metal poured into that ring. This male die when cold will not stick, but will separate readily, and from the same female die I usually make three or four male dies, numbering them in their order as taken.

I begin swedging upon the one last taken, leaving the first male die, which is, of course, the most accurate for the final swedging.

This method, though possibly somewhat more laborious than some others, produces, in my opinion, emphatically, the most perfect results.

Adjourned.

S. E. Davenport, D.D.S., M.D.S.,
*Editor of The New York Institute of Stomatology.*

## Figures and Tables

**Fig. 1. f1:**
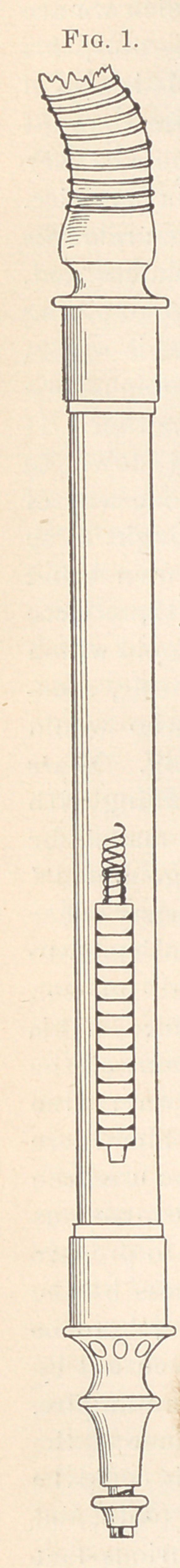


**Fig. 2. f2:**
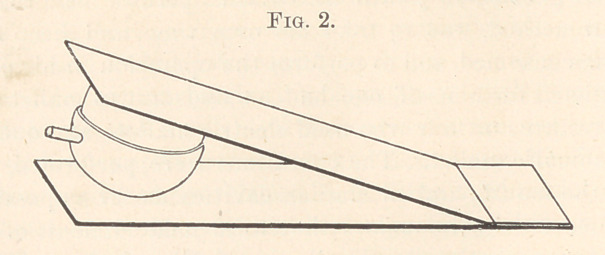


**Fig. 3. f3:**